# From Blood to Bone—The Osteogenic Activity of L-PRF Membranes on the Ex Vivo Embryonic Chick Femur Development Model

**DOI:** 10.3390/ma14247830

**Published:** 2021-12-17

**Authors:** Inês Francisco, Francisco Vale, Victor Martin, Maria Helena Fernandes, Pedro Sousa Gomes

**Affiliations:** 1 Institute of Orthodontics, Faculty of Medicine, University of Coimbra, 3004-53 Coimbra, Portugal; 2 Laboratory for Bone Metabolism and Regeneration, Faculty of Dental Medicine, University of Porto, 4200-393 Porto, Portugal; victorzmartin@gmail.com (V.M.); mhfernandes@fmd.up.pt (M.H.F.); pgomes@fmd.up.pt (P.S.G.); 3 LAQV/REQUIMTE, University of Porto, 4160-007 Porto, Portugal

**Keywords:** platelet-rich fibrin, bone transplantation, bone regeneration, angiogenesis modulating agents, chorioallantoic membrane

## Abstract

(1) Background: To evaluate the effects of the direct and indirect contact of leukocyte and platelet-rich fibrin (L-PRF) on bone development, in an ex vivo embryonic chick femur model. (2) Methods: Both sections of L-PRF membranes (red and yellow portions) were evaluated with scanning electron microscopy and histochemical staining. The in vivo angiogenic activity was evaluated using a chorioallantoic membrane model. The osteogenic activity was assessed with an organotypic culture of embryonic chick femora through direct and indirect contact, and assessment was conducted by microtomographic and histological analysis. Descriptive statistics, One-Way ANOVA and Tukey’s multiple comparisons tests were performed for datasets that presented a normal distribution, and Kruskal-Wallis tests were performed for non-parametric datasets. A significance level of 0.05 was considered. (3) Results: The L-PRF induced angiogenesis reflected by a higher number and a larger and more complex gauge in the vessels that invaded the membrane. The physical presence of the membrane over the bone (direct contact) unleashes the full potential of the L-PRF effects on bone growth enhancement. The greatest increase in mineral content was observed in the diaphysis region. (4) Conclusion: The L-PRF direct contact group presented higher values on mineral content for bone volume, bone surface and bone mineral density than the indirect contact and control groups.

## 1. Introduction

Bone loss can lead to changes in support and form, as well as the loss of metabolic functions of the bone tissue. Within the oral-maxillofacial complex, alveolar bone regeneration is often required to manage bone defects, which can occur due to several factors, such as congenital defects, alveolar bone resorption, trauma, neoplasm resection and bone infection [1,2].

Currently, various types and forms of bone graft are accessible (autogenous bone, allograft and bone graft substitutes) and can be successfully applied in a clinical scenario, with different properties of osteogenesis, osteoinduction and osteoconduction [3]. The autologous bone graft is currently considered the gold standard, due to the osteoconductive and osteoinductive properties combined with histocompatibility. Despite these advantages, it also presents several limitations, such as donor site morbidity, limited donor supply and increase of operative time. Therefore, other forms of bone graft have been proposed, also presenting limitations, such as limited immunological compatibility and biological response. Moreover, the use of bone grafts alone requires a long healing process, which may lead to some rate of relapse (bone resorption can reach up to 40%), compromising the clinical outcome and increasing the need for reintervention [1,4]. Thus, more recently, the concomitant use of natural- or synthetic-derived growth factors has been proposed to enhance the biological response, namely, by increasing the bone healing [5,6].

Platelet derivatives are a promising source for autologous regenerative therapies, given their ability to release cytokines and growth factors, modelling the immune-inflammatory response and potentially improving soft and hard tissue healing and regeneration. At present, one of the most widely used platelet concentrates is platelet rich fibrin (PRF). Since its introduction by Choukroun et al., several modifications in the protocol have been reported. Shah et al. summarized the different PRF protocols available in the literature, namely, leukocyte and platelet-rich fibrin (L-PRF), advanced platelet rich fibrin (A-PRF), advanced platelet rich fibrin + (A-PRF+) and injectable platelet rich fibrin (I-PRF) [7]. The leukocyte and platelet-rich fibrin is a second-generation fibrin-rich platelet concentrate prepared by blood centrifugation without thrombin or anticoagulants, which results in the formation of three distinct layers: platelet-poor plasma at the top; L-PRF in the middle and red blood cells at the bottom of the tube [8,9]. This concentrate contains high levels of distinct factors, such as the vascular endothelial growth factor (VEGF), the transforming growth factor-beta1 (TGF-β1), the platelet-derived growth factor (PDGF) and the interleukins (IL)-1β, IL-4 and IL-6, which promote the proliferation and differentiation of fibroblasts, endothelial cells, osteoblasts and chondrocytes, inducing tissue regeneration [8,9,10]. PRF bioactive levels are maintained for a period of 28 days due to physiological polymerization, which allows cytokines and growth factors to be stored and slowly released later [11]. The architecture of L-PRF creates a supportive scaffold, which can assist the stabilization of implanted bone grafts and enhance cell migration and angiogenesis [12]. Its angiogenesis effect, mediated by VEGF, PDGF and the basic-fibroblast growth factor, stimulates the expression of αvβ3 integrin, which will promote the linking of endothelial cells to fibrin, vitronectin and fibronectin, further enhancing the scaffolding functionality. Likewise, its relative availability and ease of preparation, cost effectiveness and bio-functionality allows its potential application in distinct regenerative applications [7]. Additionally, the presence of leukocytes might prevent infection and regulate the inflammation process [13].

Despite the promising characteristics of L-PRF that may potentially assist with soft and hard tissue healing/regeneration, previous studies have shown contradictory results [14,15,16]. Lee et al. reported that after two months of follow-up, the L-PRF without associated bone graft material failed to enhance bone formation, as compared to the unfilled control group [17]. Rexhepi et al. found that L-PRF or collagen membrane combined with inorganic bovine bone graft presented a similar effect on clinical attachment level in the treatment of infrabony defects, but L-PRF showed more radiographic defect bone level gain [18]. Al-Mahdi et al. evaluated sixteen patients and reported that the L-PRF group (L-PRF mixed with an autogenous bone graft) increased bone density after 6 months and significantly reduced postoperative bone resorption compared with the control group [6]. Conversely, Omidkhoda et al. showed no significant differences in the density and height of maxillary alveolar cleft reconstruction at three months with L-PRF [16]. These distinct findings may be due to the lack of standardization across the studies regarding the protocol of L-PRF preparation, outcome measurement methods (two or three dimensional image diagnostic tool or unit of measurement–cubic centimeter or percentage of newly formed bone) and follow-up times (which varies from days to months). On the other hand, it must be pointed out that the current evidence comes from in vitro, in vivo and clinical studies with small sample sizes [6,7,8,9,10,11,12,13,14,15,16,17,18,19,20]. Thus, the heterogeneous results present in the literature reinforce the need for an experimental approach with a representative model to validate the potential effects of L-PRF-mediated osteogenic modulation.

The ex vivo culture model allows the manipulation of cells and tissues in a spatial arrangement comparable to in vivo models, avoiding the systemic influences. Moreover, this model is cost-effective and avoids ethical issues present in animal experimentation. The ex vivo embryonic femur chick model is an established model system for evaluating bone tissue-engineering strategies, given the high responsiveness to external stimuli, the absence of an immune system in early stages of development and the biological translationality to the bone development and functionality of mammals [21,22].

Therefore, this study aims to assess the L-PRF modulation capability, either directly or indirectly, on the bone development of the embryonic chick femur, grown ex vivo.

## 2. Materials and Methods

### 2.1. L-PRF Membrane Preparation

Peripheral blood samples were collected into four sterile vacutainer tubes (9 mL), without bovine thrombin or anticoagulants, from a healthy human volunteer, after written informed consent was obtained (Ethics Committee of Faculty of Medicine of University of Coimbra approval, number CE-049/2019). The samples were immediately placed symmetrically into the centrifuge device (IntraSpin™, Intra-Lock, Boca Raton, FL, USA), being subjected to the centrifugation process (2700 rpm for 12 min). Afterwards, the fibrin clots were separated from the red blood cells and placed in an Xpression^TM^ box for compression by gravity. Finally, clots were carefully separated into four sections using a scalpel blade, with sections being approximately 3 mm long and 1 mm wide.

### 2.2. Characterization of L-PRF Membranes

L-PRF membranes were observed by scanning electron microscopy (SEM). For this, membranes were fixed (1.5% glutaraldehyde, 30 min), dehydrated in graded alcohols, critical-point dried, sputter-coated with gold and analysed in a scanning electron microscope equipped with X-ray energy dispersive spectroscopy (EDS) microanalysis capability (Quanta 400FEG ESEM/EDAX Genesis X4M, FEI, Hillsboro, OR, USA).

In addition, L-PRF membranes were assessed with haematoxylin and eosin (H&E) stain and Masson’s trichrome histochemical stain. Briefly, L-PRF sections were fixed in formaldehyde 4% and then embedded in paraffin blocks for thin sectioning. After deparaffinisation and rehydration, sections were stained with Wiegert’s haematoxylin and counterstained with eosin. Alternatively, sections were stained with the Trichrome Stain (Masson) Kit (Sigma-Aldrich, Saint Louis, MO, USA) [23].

### 2.3. In Vivo Angiogenic Activity of L-PRF

The angiogenic activity of L-PRF was evaluated using the chorioallantoic membrane (CAM) model [24]. The assay was performed according to Schmitd et al., with some modifications [25]. In short, fertilized chicken eggs (Gallus domesticus) were incubated in an Octagon 40 ECO rotating egg incubator (Brinsea, UK) at 37.5 °C, 50% humidity and one rotation per hour, for 8 days. The incubation was interrupted, and the air sac was delimited. The eggshell from the marked region was carefully removed, as well as the outer membrane of the egg. A section of the L-PRF membrane (5 × 10 mm) was placed on the exposed CAM and the egg was covered with a sterile transparent film. The eggs were then incubated in the same conditions for 2 days, without rotation. Finally, the CAM was removed with the L-PRF membrane and fixed with 4% formaldehyde for 4 h. The CAM was imaged with a Zeiss Stemis 305 stereo microscope. Images were captured using an Axiocam 5 Colour Camera (Zeiss, Jena, Germany). For the average vascular diameter measurement, the Vessel Analysis plugin of the Fiji software (v.1.53c) was applied [26]. Experiments were performed in triplicate and representative macrographs were acquired for all the conditions.

### 2.4. Ex Vivo Osteogenic Activity of L-PRF

The osteogenic activity of the L-PRF was assessed by its incubation with an organotypic culture of embryonic chick femora. Fertilized chick eggs (Gallus domesticus) were maintained in an Octagon 40 ECO rotating egg incubator (Brinsea, UK) in the following conditions: 37.5 °C and 50% humidity, for 11 days. Following this, embryos were euthanized, and the muscles and soft tissues of the femora were carefully removed. Subsequently, whole femora were washed in saline solution and placed into Netwell^TM^ Inserts (440 µm mesh size polyester membrane, 30 mm diameter, Corning), in six-well–plates (Costar® 6-well Clear TC-treated Multiple Well Plates, Corning reference 3516). Experimental conditions were defined according to the following groups: (i) femora grown in direct contact with L-PRF membrane; (ii) femora grown in indirect contact with L-PRF membrane, under the Netwell^TM^ insert at the bottom of the culture plate and (iii) femora grown in the absence of L-PRF membrane (control). L-PRF membrane sections (dimensions 5 × 10 mm) were used for the assay, which was performed in sextuplicate. The basal culture medium (1 mL/well) consisted of Minimum Essential Medium Eagle (α-MEM) with ascorbic acid 2-phosphate (50 µg/mL), penicillin (100 U/mL) and streptomycin (100 µg/mL). Femora were kept for 11 days in the basal culture medium, at the liquid/gas interface, in a humidified atmosphere of 5% CO2 in air and 37 °C. Culture media were changed daily. On day 11, cultured femora were washed in phosphate buffered saline, fixed and processed for microtomographic (μCT) and histological analysis.

#### 2.4.1. Microcomputed Tomography (μCT)

Femora were fixed and stored in a 70% ethanol solution at room temperature. μCT analyses were performed using a SkyScan 1276 scanning system (Bruker micro-CT, Kontich, Belgium) with the following parameters: 4.5 μm pixel size, 40 kV X-ray voltage, source current 100 µA and an exposure time of 800 ms. The sample containers (1.5 mL Eppendorf tubes) were imaged using a detector assembly over a 360° sample rotation (*n* = 4).

The reconstruction and the correction of beam hardening, ring artefacts, smoothing and misalignment parameters was performed by NRecon software v.1.7.4.2. In addition, CTAnalyser software (v.1.17.7.2 Bruker micro-CT, Kontich, Belgium) was used to select a volume of interest (VOI), defined as 2 mm in the proximal and distal directions from the mid-diaphysis, with a total of 900 layers. The following histomorphometric parameters were calculated: bone volume (BV), tissue volume (TV), bone surface (BS) and bone mineral density (BMD). Three-dimensional images were taken using CTVox software (Brucker, Kontich, Belgium, version 3.3.0).

#### 2.4.2. Histochemical Assessment

Femora were fixed using formaldehyde 4% and embedded in paraffin blocks. After sectioning, samples were deparaffinized, hydrated, and stained in Alcian blue and Picrosirius red solutions [27]. Samples (*n* = 4) were observed using a Zeiss Axiolab 5 microscope and images were captured using an Axiocam 5 Colour Camera (Zeiss).

### 2.5. Statistical Analysis

All analyses were performed using the Statistical Package for the Social Sciences, version 26.0 for Windows (SPSS Inc., Chicago, IL, USA). A significance level of 0.05 was considered. Descriptive statistics were obtained using mean and standard deviation values. A Shapiro-Wilk normality test was performed for each variable, followed by One-Way ANOVA and Tukey’s multiple comparisons tests for datasets that presented a normal distribution, or Kruskal-Wallis test for non-parametric datasets.

## 3. Results

**Figure 1:**
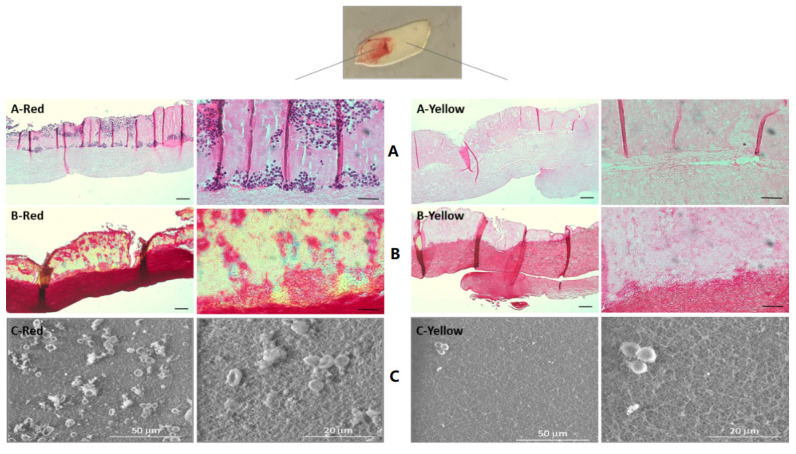
Microscopic appearance of the L-PRF membrane with its distinct portions. Images on the left side correspond to the red portion, while images on the right correspond to the yellow portion. (**A**) Haematoxylin and eosin (H&E) staining: fibers were stained in pink and the cellular nuclei were stained in purple. (**B**) Masson’s trichrome staining, fibers and erythrocytes were stained in bright red and nuclei in black. Scale bar = 100 and 50 µm respectively. (**C**) SEM images of the L-PRF membrane. High magnification images showed the porous morphology of the fibrin strands network. The red portion of the membrane contains a high number of erythrocytes and platelets. Attached platelets showed abundant thin cytoplasm extensions suggesting an activated state.

L-PRF membranes were characterized into red and yellow portions based on their morphological aspects. The H&E staining (Figure 1A) showed that both sections were composed of a dense fibrin clot, with a minor degree of porosity within the organized fibers. However, the red portion (Figure 1A-Red) presented several blue-stained nuclear leukocytes, distributed across the upper region of the L-PRF, which were rarely identified in the yellow portion (Figure 1A-Yellow). In addition, the fibrin clot at the upper layer of the red portion displayed a more intense red/pinkish accent, suggesting the presence of erythrocytes among the leukocytes.

A similar trend was observed in the Masson’s trichrome staining (Figure 1B): the organisation of both portions was similar, despite the differences on cellular composition and the fibrin’s density. The red portion (Figure 1B-Red) presented a layer with a yellowish accent, a faint and dispersed dark color and numerous red nodes, pointing out the presence of cells, which were more rarely identified in the yellow portion (Figure 1B-Yellow). Further, the bottom layer of the red portion presented an intense red staining, while the yellow portion presented a less dense staining associated with increased inter-fibrillar spaces. These findings corroborate those of the H&E staining, in which the red portion presented an upper layer with entrapped platelets and cells and a bottom layer made of a dense fibrin clot, while the yellow portion presented a less dense fibrin clot and rarely distributed cells. SEM images (Figure 1C) confirmed the porous morphology of the L-PRF, resulting from the fibrin strands network. The red portion of the membrane contained abundant clusters of red blood cells, platelets and leukocytes, as compared to the yellow portion. Moreover, these attached platelets of the red portion displayed abundant thin cytoplasm extensions, suggesting an activated state.

**Figure 2:**
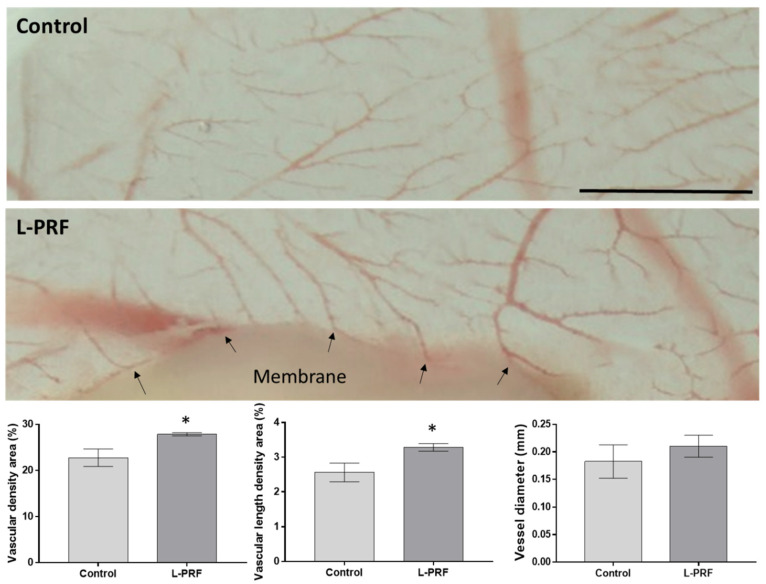
The angiogenic assessment of L-PRF using a CAM model in the absence (control) or presence of L-PRF membranes, in the red portion and the yellow portion. The L-PRF was grafted onto the CAM and the eggs were incubated at 37 °C for 2 days. It is noticeable that there was increased vessel sprouting into the membrane structure (arrows), as well as an increased average diameter of the vessels in the presence of the L-PRF, corroborated by the quantitative analysis of vascular density area, vascular length density area and vessel diameter, demonstrated as graphs below the images. Scale bar corresponds to 2 mm.* Statistically significant, *p* < 0.05, *n* = 3.

The in vivo angiogenic potential of the L-PRF was assessed by the CAM assay (Figure 2). In the control group, numerous thin vessels arranged in a network were observed dispersed throughout the CAM structure, in addition to few thicker vessels. When in contact with the L-PRF for 48 h, a vascular network constituted by thicker vessels was verified, particularly in the L-PRF vicinity, further presenting a more complex and tortuous organization. The quantitative analysis confirmed the increase in vascular density areas in the experimental group, attaining statistical significance, as well as an upward trend in the vessel’s diameter. Besides this, vessel sprouting into the L-PRF structure could be identified.

The osteogenic potentiality of the L-PRF was assessed within an ex vivo embryonic chick femur development model. Femora were characterized in the presence of L-PRF, either in direct or indirect contact, through microtomographic and histological/histochemical analysis.

**Figure 3:**
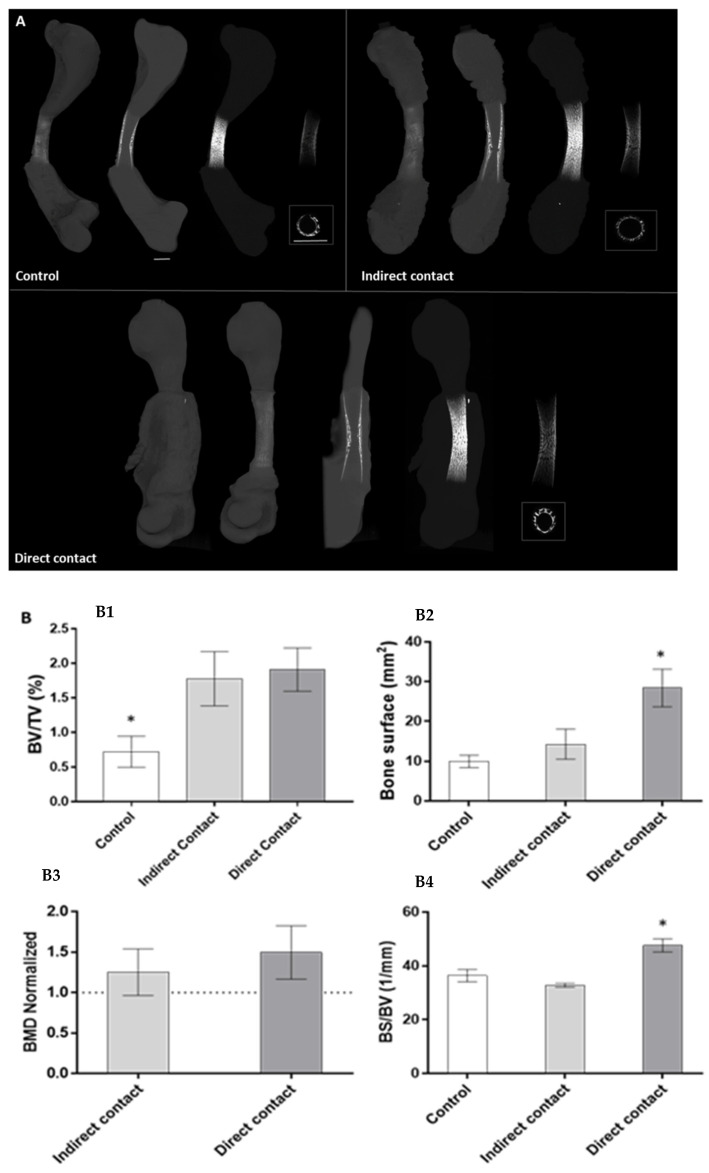
Microcomputed tomography analysis of D11 chick femora following 11-day culture in the absence (control) or presence of L-PRF membranes, in indirect and direct contact. (**A**)—Three-dimensional representative images of samples (whole femur, sagittal section and maximum intensity projection of the whole femur, sagittal/ cross-section of the central diaphysis region, respectively) of the distinct experimental conditions. Scale bar corresponds to 500 µm. (**B**)—Quantitative morphometric parameters of the samples: (**B1**)—BV/TV; (**B2**)—BS; (**B3**)—BMD; (**B4**)—BS/BV). BMD was normalized to the control values, corresponding to 1 unit. * Statistically significant, *p* < 0.05, *n* = 6.

Figure 3A illustrates the differences regarding mineralization among the groups. It is noticeable that the control group presented the smallest mineralized area, as well as a dimmer brightness in the diaphyseal region. Comparatively, the experimental L-PRF groups, either in direct or indirect contact with femora, presented an increased and denser mineral layer at the diaphysis region. In addition, the quantitative microtomographic differences regarding bone volume, tissue volume, bone surface, bone surface/bone volume ratio and bone mineral density within the defined ROI are displayed in Figure 3B. The control group presented the lowest values for the assessed parameters. The indirect contact L-PRF group showed a trend for increased levels in relation to the control group, attaining statistical significance in the BV/TV ratio. The direct contact L-PRF group presented the highest values, with significantly higher levels for all the assayed parameters.

**Figure 4:**
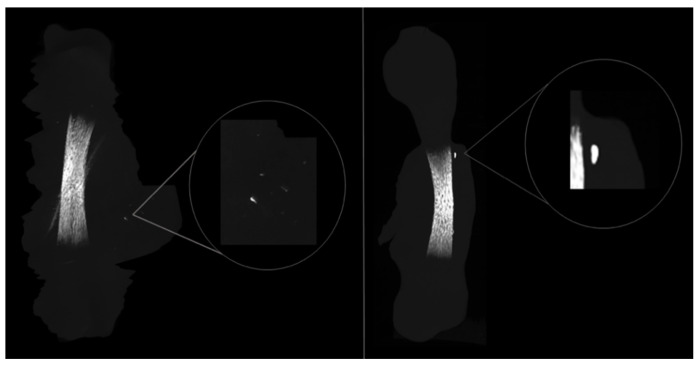
Representative three-dimensional microcomputed images of bone nodules found across the L-PRF membrane in the direct-contact experimental group, emphasising the osteogenic potential of the L-PRF membrane.

Moreover, during the ROI demarcation for the microcomputed tomographic analysis, mineralized nodules were found within L-PRF membranes in the direct contact group, illustrated by Figure 4. Those nodules were located close to the diaphysis region and were completely decoupled from the femora, suggesting an ectopic mineralization within the structure of L-PRF membrane.

**Figure 5:**
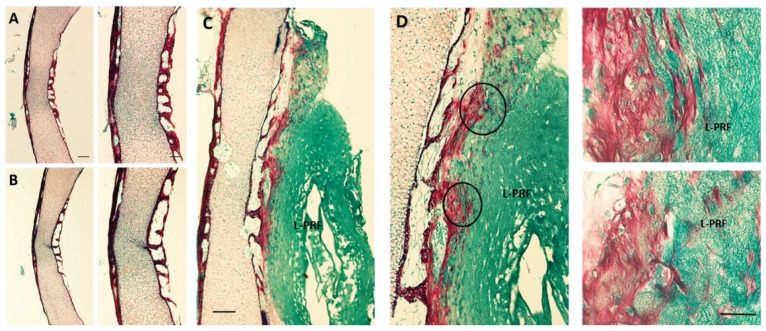
Representative histological images (Alcian blue/Sirius red stain) of the diaphyseal region of ED11 chick femora, after 11 days of organotypic culture without and with L-PRF membrane, in indirect and direct contact. (**A**) Control group, (**B**) indirect contact group and (**C**) direct contact group. Scale bar = 100 and 50 µm, respectively. (**D**) Represents magnified areas from the marked areas, scale bar = 20 µm.

In order to complement the imagiological data, the L-PRF osteogenic potentiality was assessed through a histochemical staining, using Alcian blue to dye the proteoglycan-rich cartilage and Sirius red to dye the collagen content, presumably associated with the osteogenic matrix (Figure 5). The control group (Figure 5A) exhibited a deposition of the collagenous matrix in red, at the peripheral region of the mid-diaphysis, with a central portion stained with a blueish accent, evidencing the proteoglycan-rich layer. In addition, the outer-layer presented a trabecular-like morphology, characteristic of the ongoing osteogenic process. In comparison, the indirect-contact group (Figure 5B) presented no significant differences, apart from minor variations in the outer layer organization, corroborating the µCT analysis. Regarding the direct contact group (Figure 5C), the differences were more noticeable: the L-PRF was deeply stained in blue and appeared to be merged with the femoral bone surface, while the diaphysis maintained the morphological arrangement and staining previously observed in the other groups. However, the collagenous-rich layer seemed to be thicker, and an increased and denser red stain could be observed from the trabecular organization established at the peripheral region of the bone, growing centrifugally into the L-PRF membrane.

## 4. Discussion

This study aimed to characterize the L-PRF membrane and investigate its angiogenic potential using an in vivo CAM model, as well as its osteogenic activity on the chick embryo femur model, assigning this ex-vivo system as a viable alternative method to further studies on L-PRF-mediated bone development and regeneration. 

Regarding the characteristics of the L-PRF membrane (Figure 1), both the red and yellow portions were found to have the characteristic arrangement of dense fibrin fibers with tight junctions, containing mononuclear leukocytes and red blood cells, as described in the literature [28]. In addition, the topography observed was similar, as described in the study by Fujioka-Kobayashi et al., in that both sections presented a rough surface, with leukocytes, platelets and crushed red blood cells interspersed with the fibrillar matrix [29]. Comparatively, the red portion presented an increased amount of leukocytes and red blood cells. Indeed, several studies have suggested that the red portion may have a more positive effect on bone regeneration than the yellow portion [30,31]. Ehrenfest et al. found that the red portion included a higher number of growth factors and cytokines, while the yellow portion allowed for the delivery of a fibrin gel serving as a structural support material [31]. More recently, Thanasrisuebwong et al. reported that the red portion had a greater effect on cell proliferation and cell migration, but a lower fibrin network density. This finding may be explained by the high content of platelets and cells present on the red portion, which can release more growth factors, consequently inducing cell recruitment and growth [30]. These findings highlight that knowledge of the L-PRF architecture is essential in order to make reasonable use in different clinical situations. Both portions of L-PRF may have an important role in bone regeneration. The red portion may function as a reservoir for cells and growth factors that enhance cell proliferation and migration, while the yellow portion expectedly allows the controlled release of these cells/growth factors and serves as a support interface for bone grafts due to its dense fibrin structure. Moreover, L-PRF promotes the release of leukocytes, which might regulate the inflammation process, contributing to improving the prognosis of future prosthetic rehabilitation that patients may require, for example, reducing the risk of peri-implantitis [13]. Öncü et al. found that L-PRF can promote faster osseointegration and bone-implant contact due to the increase in the percentage of new bone formation during the initial healing stage [32].

The angiogenic potential of the L-PRF was evaluated using the CAM assay (Figure 2). Due to the presence of angiogenic-growth factors in the L-PRF, such as VEGF, increases in vascular density, complexity and tortuosity were expected, as described by Talavera-Adame et al. [24]. Those parameters can be noted in the present study—apparently, CAM in contact with the L-PRF presented more vessels, with an augmented diameter, more ramifications and some vascular linkage with the membrane. Further, an increase in the vessel’s diameter of approx. 15% was confirmed by the quantitative analysis. In fact, the study by Kobayashi et al. analysed L-PRF preparations using the CAM assay, which resulted in a significant increase in the number of blood capillaries, as well as the amount of mature blood vessels [33]. Similar results can be observed in the study of Ratajczak et al.—L-PRF membranes resulted in more blood vessels in the CAM compared with untreated eggs. Furthermore, a significant increase in proliferation and cell migration was observed when in vitro cultures of human umbilical vein endothelial cells were incubated with L-PRF leachates [34].

The success of bone regeneration depends on two main factors: a structural support material that allows neovascularization and cell recruitment and a matrix that delivers morphogenetic, regulatory and growth factors that orchestrate the biological response [35]. This study showed that the L-PRF groups, in either direct or indirect contact with the growing bone, induced higher values for bone volume, bone surface and bone mineral density than the control. This result is in accordance with the systematic review by Castro et al. who showed that the application of L-PRF alone promotes less buccal bone resorption compared to natural healing, resulting in the preservation of the alveolar width [36]. Additionally, the positive effect of adding L-PRF to particulate bone graft material has been reported by several studies [35,36,37]. Knapen et al. found contradictory results. According to these authors, L-PRF does not have any additional effect on bone regeneration, since no differences within the four studied groups (L-PRF, bovine hydroxyapatite (BHA), BHA + L-PRF, and control) were found regarding bone quality and quantity [38]. Therefore, the direct interaction between platelet-rich fibrin and osseous cells in the healing process is insufficiently documented, presenting contradictory results [12,13,14,15,16]. The distinct results between studies may be caused by the different protocols used regarding L-PRF preparation or regarding experimental protocols (with diverse study models, surgical procedure and characterization methodologies). Moreover, Ehrenfest et al. reported that L-PRF is a nonhomogeneous biomaterial that presents localized differences since it is constituted by a heterogeneous geographical cellular distribution [39]. Therefore, it is expected that the position of the L-PRF membrane will also interfere with the results obtained, since it may lead to a varying input from growth factors. This study verified this assumption because the physical contact of L-PRF over the bone showed the best results on bone growth enhancement, while the indirect contact showed a beneficial but limited effect. The direct contact group appears to perform the direct mobilization of growth factors and cytokines during cell migration, which allows more retention of these factors compared to the indirect contact group, optimizing the effects of tissue regeneration.

Furthermore, the osteogenic response of the direct contact group occurred mostly in the diaphysis region (Figure 3), and a thicker collagen outer-layer was highlighted by the histochemical staining (Figure 5C) in comparison with the control and the indirect contact groups, representing the mineralized and non-mineralized osteoid matrix [27]. These results are in line with the study by Shawky et al. that studied the effect of PRF in the quality and quantity of unilateral maxillary alveolar clefts, reporting a higher mean amount of newly formed bone volume in the PRF group [37]. In addition, mineralized nodules within the L-PRF membrane were found in the µCT analysis (Figure 4). Aligned with these findings, some collagenous compositions (red stained) were also noted within the L-PRF membrane in the histochemical staining images (Figure 5C), suggesting progressive cellular outgrowth and osteogenic differentiation with the deposition of a collagenous-rich matrix. This was an unexpected result because Knapen et al. reported that connective cells could be seen in the osteotomy region after the application of L-PRF without extending into the L-PRF membrane [38]. The osteogenic induction found by the results of this study may be related to the presence of several growth factors in the L-PRF, namely, the fibroblast growth factor, which induces pre-osteoblasts differentiation and proliferation; VEGF, which enhances angiogenesis and promotes a mitogenic activity and synergy between PDGF with TGF-β, which contributes to the proliferation of cells (marrow stem cells, fibroblasts, pre-osteoblasts) [40]. It is also of note that, in the present experimental model, the L-PRF membrane was placed in direct contact with periosteum, an environment rich in osteoblastic-precursor populations with a high regenerative potential, given the increased clonogenicity, proliferation and osteogenic differentiation capability as compared with bone marrow stromal cells/skeletal stem cells, which may further sustain the enhanced osteogenic outcome [41].

Previous studies faced great difficulties regarding PRF research in animal models due to several reasons, namely limited blood volume, variations in experimental design and differences in biology and physiology. This ex vivo model encountered several limitations, namely the combination of L-PRF human samples with chick femurs. However, as this study used femora in early development (embryonic day 11), it did not have yet an immune system [21]. Additionally, L-PRF is used locally and not systematically, which reduces the risk of immunologic reactions and toxic risks.

Despite these limitations, this study presents a viable model for assessing the effects of L-PRF on bone regeneration. The present model is more cost-effective and avoids several of the disadvantages of in vivo models, namely bias in data obtained due to systematic influences, large numbers of animals and ethical issues present in animal experimentation. However, further studies should verify the potential of clinical translation. The association effect of L-PRF and bone graft (autogenous bone, allograft and bone graft substitutes) on the bone healing defects, as well as the potential effect of L-PRF on bone regeneration in systemic diseases such as osteoporosis and diabetes, should be investigated.

## 5. Conclusions

The ex vivo chick femur is a viable model for assessing the effects of L-PRF on bone regeneration. The L-PRF direct contact group presented higher values regarding mineral content for bone volume, bone surface and bone mineral density in comparison with the L-PRF indirect contact and control groups.

## Data Availability

The data presented in this study are available on request from the corresponding author.
